# Patient‐reported outcomes and visual function after four refractive strategies in cataract surgery: A randomized controlled trial

**DOI:** 10.1111/aos.70145

**Published:** 2026-04-13

**Authors:** Anne Guldhammer Skov, Morten la Cour, Birgitte Moldow, Lars Holm, Therese Krarup

**Affiliations:** ^1^ Department of Ophthalmology Copenhagen University Hospital—Rigshospitalet Copenhagen Denmark; ^2^ Faculty of Health Sciences University of Copenhagen Copenhagen Denmark

**Keywords:** cataract, cataract surgery, patient satisfaction, patient‐reported outcome measures, refractive strategies

## Abstract

**Purpose:**

To evaluate patient‐reported outcome measures (PROMs) and visual function across four groups of public cataract patients: emmetropia, mini‐monovision, monovision with partial‐range of field (RoF) narrow intraocular lenses (IOLs) and patients implanted with partial‐RoF extended IOLs.

**Methods:**

This randomized controlled trial included consecutive patients with bilateral symptomatic cataract randomized to implantation of partial‐RoF narrow IOLs targeting (1) emmetropia (both eyes), (2) mini‐monovision (emmetropia in the dominant eye, −1.25 diopters (D) in the non‐dominant), (3) monovision (emmetropia in the dominant eye, −2.50 D in the non‐dominant) or (4) implantation of partial‐RoF extend IOLs (+0.01 to +0.25 D in the dominant eye, −0.01 to −0.25 D in the non‐dominant eye). Assessment included four PROMs, binocular uncorrected distance, intermediate and near visual acuity (VA), aniseikonia, stereoacuity and safety.

**Results:**

188 patients were randomized to the four groups. High levels of improvement in postoperative PROM scores were observed across all groups, with no significant differences between groups. All patients achieved binocular best corrected distance VA of logMAR 0.12 or better (range 0.12 to −0.26). Mini‐monovision and partial‐RoF extend yielded comparable outcomes across visual performance, spectacle independence and safety. Monovision provided superior uncorrected near VA and the lowest spectacle use but was associated with increased aniseikonia, impaired stereoacuity, worse binocular uncorrected distance VA, more frequent halos, lower recommendation rates and a 6.7% reoperation rate for binocular visual complaints.

**Conclusion:**

All four groups achieved high levels of improvement in visual function and PROM scores. There was no statistically significant difference in PROM scores between the groups.

## 1 INTRODUCTION

Advancements in cataract surgery have raised patient expectations regarding postoperative visual outcomes, including spectacle independence. To meet these demands, intraocular lens (IOL) technology has evolved, offering a wide range of optical designs and functionalities. IOLs can be classified into two main categories based on the best distance corrected monocular visual acuity (VA) defocus curves' range of field (RoF) and shape: partial‐RoF (including partial‐RoF narrow and partial‐RoF extend IOLs) and full‐RoF (including full‐RoF continuous IOL) (Ribeiro et al., 2024). These IOLs differ substantially in optical principles and functional performance. Generally, full‐RoF continuous IOLs provide high uncorrected VA at all distances and high spectacle independence but are associated with a higher incidence of glare, halos or starbursts (Calladine et al., 2012; de Silva et al., 2016; Greenstein & Pineda II, 2017; Khandelwal et al., 2019; Lyra et al., 2025), whereas most partial‐RoF extend IOLs offer good uncorrected distance and intermediate vision but reduced near vision, and a lower frequency of halos and glare (Lesieur & Dupeyre, 2023; Nowik et al., 2022).

Monovision represents an alternative refractive approach to achieve spectacle independence, achieved by implanting partial‐RoF narrow IOLs, targeting one eye for distance and the fellow eye for near vision. The main disadvantages of monovision are the risk of clinical aniseikonia. A preoperative monovision contact lens trial is often performed to evaluate patient tolerance prior to surgically induced anisometropia. However, this approach is time‐consuming, requires multiple clinical visits and may be unreliable in cataract patients due to the reduced vision caused by the cataract. Previous studies have demonstrated substantial interindividual variability in tolerance to anisometropia (Greenbaum, 2002; Ito et al., 2009; Krarup et al., 2020). Despite its widespread use, evidence regarding tolerance thresholds and the optimal degree of anisometropia in terms of patient satisfaction remains limited (Skov et al., 2026).

In public hospitals, cataract surgery is primarily performed to restore safe and comfortable vision in daily life rather than to complete spectacle independence. In this elderly population, many patients may mainly seek improved functional vision for everyday activities, such as reading road signs or navigating safely. Nevertheless, reducing dependence on spectacle—especially for intermediate and near tasks—may significantly enhance postoperative visual function and patient satisfaction. However, whether spectacle independence confers additional benefit in visual function in a population already experiencing marked visual improvement following surgery remains unknown.

Traditionally, VA has served as the primary clinical outcome when evaluating cataract and refractive surgery (de Silva et al., 2016). However, VA alone may not fully reflect the subjective patient experience. Consequently, patient‐reported outcome measures (PROMs) have gained increasing importance for assessing postoperative outcomes (Goldberg, 2003; Ito et al., 2009; Rutstein et al., 2015; Zhang et al., 2015). Although numerous PROMs exist, no standardized consensus currently defines assessment methodology. The Catquest and the Quality of Vision (QoV) questionnaire are Rasch‐validated tools and are widely used for evaluating outcomes after cataract surgery (Alió Del Barrio et al., 2025; Galvis et al., 2022; Khadka et al., 2016; Lundstrom et al., 2011; Nielsen et al., 2016, 2025; Rein et al., 2025).

In this randomized clinical trial, we evaluated changes in PROMs and visual function following four refractive strategies in public cataract surgery—emmetropia, mini‐monovision, monovision with partial‐RoF narrow IOLs and partial‐RoF extend IOLs—and examined whether the three strategies aimed at reducing spectacle dependence provide additional value compared with emmetropia, beyond the visual improvement achieved through cataract surgery.

## 2 METHODS

### 2.1 Study design and approvals

We conducted a single‐center, randomized controlled pilot trial to evaluate four refractive strategies. Patients underwent same‐day bilateral phacoemulsification and were randomized (1:1:1:1) to: (1) emmetropia (emmetropia in both eyes (first minus)), (2) mini‐monovision (emmetropia in the dominant eye, −1.25 diopters (D) in the non‐dominant), (3) monovision (emmetropia in the dominant eye, −2.50 D in the non‐dominant) or (4) partial‐RoF extend (minimal hypermetropia (+0.01 to +0.25 D) in the dominant eye and minimal myopia (−0.01 to −0.25 D) in the non‐dominant eye).

Groups 1–3 received bilateral partial‐RoF narrow IOLs (Alcon Acrysof ACU0T0 until December 2021 and Johnson & Johnson Tecnis GCB00/ZCB00 thereafter). The change in partial‐RoF narrow IOL was due to a department‐wide transition following a mandatory national public tender.

The two partial‐RoF narrow IOLs differ slightly in the optical material: an acrylate/methacrylate copolymer (Alcon ACU0T0) versus an acrylic material (Johnson & Johnson GCB00/ZCB00), resulting in differences in the refractive index and Abbe numbers (1.55/37 versus 1.47/55) and hence in chromatic aberration. They also differ marginally in spherical aberration (−0.20 μm versus −0.27 μm).

The partial‐RoF extend group received bilateral Alcon AcrySof IQ Vivity IOLs. A partial‐RoF extend IOL was chosen over a full‐RoF continuous IOL based on its visual performance and side‐effect profile with lower halos and glare.

Ethics approval was obtained by the Regional Research Ethics Committee (H‐18058722), ensuring compliance with the principles of the Declaration of Helsinki, and registered at the Danish Data Protection Agency (VD‐2019‐194) and ClinicalTrials.gov (ID: NCT05001932, date: 08/03/2021). All patients volunteered to participate in the trial. Written informed consent was obtained from all patients prior to randomization.

Potentially eligible patients received written and oral study information at the department and, after providing initial consent, underwent a same‐day preliminary assessment. They were then granted a one‐week consideration period before providing final informed consent.

### 2.2 Participants

Patients were enrolled consecutively between August 2021 and December 2023. All patients referred to Rigshospitalet, Denmark, for cataract surgery were prescreened with the following inclusion criteria: age 60–85 years, bilateral symptomatic cataract, corneal astigmatism <1.0 D, axial length (AL) 20–27 mm, interocular AL difference <0.30 mm, anterior chamber depth (ACD) >2.3 mm, dilated pupil size ≥5.0 mm, pupil size under photopic conditions ≥2.5 mm and stereoacuity ≤480 arcsecond.

Exclusion criteria were former intraocular surgery, amblyopia, strabismus, corneal pathology, glaucoma or ocular hypertension, uveitis, exudative age‐related macular degeneration, age‐related macular degeneration with geographical atrophy, reticular pseudodrusen, epiretinal membrane, retinal vein occlusion, retinal detachment, risk of weak zonules due to pseudoexfoliation syndrome or former trauma, insulin dependent diabetes mellitus, diabetic retinopathy, dementia, lack of cooperation and commercial driving.

### 2.3 Patient‐reported outcomes

We employed four PROMs to comprehensively assess different aspects of subjective visual functions.

The Catquest‐7SF questionnaire is unidimensional and focuses on improvement in visual disability and satisfaction with vision. It is a widely used PROM when evaluating patient satisfaction and visual results after cataract surgery and has demonstrated validity and reliability as an instrument for evaluating patient outcomes following cataract surgery (McAlinden et al., 2011; Sparrow et al., 2018). It has been translated into Danish, Rasch validated, and reduced to seven items, named Catquest‐7SF (Nielsen et al., 2019).

The QoV questionnaire addresses QoV through patient‐reported visual symptoms such as glare, halos and starburst. It includes symptom frequency, severity and a bothersome scale that captures the extent to which symptoms negatively affect the patient's visual experience. It is Rasch‐validated, and a frequently used questionnaire (McAlinden et al., 2010).

The Patient‐Reported Outcome (PRO) questionnaire (MDBackline, Laguna Beach, CA, USA) assesses postoperative satisfaction with surgery, vision and spectacle use. It has been validated using covariance analysis (Hovanesian, 2018; Hovanesian et al., 2019).

The Binocular Visual Function (BVF) questionnaire addresses common complaints when experiencing impaired binocular vision such as asthenopia, double vision and dizziness. It was developed for this study to compare whether groups with anisometropia had more binocular complaints (see Supporting Information S1 for a translated version).

In this trial, we translated QoV and PRO into Danish in a two‐stage process as recommended by Fayer and Machin (2016).

For Catquest‐7SF, QoV and BVF, a delta (∆) score was calculated as the difference between the four‐month postoperative and preoperative scores. The PRO was completed only postoperatively; therefore, no delta value could be calculated. Higher Catquest‐7SF Rasch scores indicate improvement in visual function. QoV consists of 10 items each with three independently Rasch‐validated question types. Mean scores of each question type were reported, with a low score indicating low visual complaints. BVF scores were calculated as the mean of the 12 items, where a low score reflects low binocular complaints.

### 2.4 Preoperative and postoperative evaluation

All patients underwent comprehensive ophthalmic examinations at three scheduled visits: preoperatively, 1 week postoperatively and 4 months postoperatively. Additional unscheduled visits were conducted on patient request.

At all visits, the following was performed: Best corrected VA (BCVA) and uncorrected VA (UCVA) were assessed at 4 m, 70 cm and 40 cm. Distance VA was measured using the ETDRS (Early Treatment Diabetes Retinopathy Study) chart at 4 m, and near VA was measured using the logMAR near vision chart (Logarithmic VA chart 2000 ‘New ETDRS’, Inc., USA) at 70 and 40 cm. VA was recorded in logarithm to the minimal angle of resolution (logMAR) and assessed both monocularly and binocularly. Measurement of intraocular pressure (IOP) (iCare tonometer, Vantaa, Finland), slit lamp examination, dilated ophthalmoscopy, stereoacuity at 40 cm with best near correction (TNO stereo test; Laméris Ootech, Ede, The Netherlands), aniseikonia at 40 cm with best near correction (New Aniseikonia Test, NAT version 3; Handaya, Tokyo, Japan) and completion of the Catquest‐7SF, QoV and BVF questionnaires.

Preoperative assessments additionally included ocular dominance (hole‐in‐hand test), optical biometry (IOLMaster 700; Carl Zeiss Meditec, Oberkochen, Germany), wavefront aberrometry, corneal topography (iTrace; Tracey Technologies, Houston, TX, USA) and optical coherence tomography (Topcon OCT; Topcon Europe Medical BV, The Netherlands).

Postoperative assessments included the PRO questionnaire (at 1 week and 4 months). At the 4‐month visit, contrast sensitivity was assessed using the Pelli–Robson Chart (Precision Vision LaSalle, IL, USA) at 1 m, with patients wearing their own optimal correction. The chart was evenly illuminated (approximately 85 cd/m^2^). The patient's sensitivity was determined by the faintest triplet in which two out of the three letters were correctly identified, as recommended by Pelli et al. (1988), and lastly an OCT imaging was performed.

Refraction was assessed objectively using a Retinomax (Right MFG Co., Ltd., Tokyo, Japan) handheld autorefractor (average of five readings) and subjectively using a trial frame, trial lenses and a cross cylinder. A near addition of +2.50 D was initially used to assess reading vision and adjusted as needed. Subjective refraction was obtained monocularly and binocularly and was used as the true refraction.

Horizontal and vertical aniseikonia were quantified, and a mean total aniseikonia was computed as the geometric mean (Krarup et al., 2020): $\sqrt{\text{mean}\;{\text{aniseikonia vertical}}^{2}+\text{mean}\;{\text{aniseikonia horisontal}}^{2}}$.

### 2.5 Randomization

Randomization was stratified by age (60–69 years/70–85 years) and gender, using block randomization with block size of 4 and 8 patients. Patients were unaware of their randomization group until final follow‐up. Randomization was based on a computer‐generated allocation sequence list using the randomization instrument in Research Electronic Data Capture (REDCap) hosted in the Capital Region, Denmark (password‐protected) (Harris et al., 2009, 2019). The list was created using https://sealedenvelope.com/simple‐randomiser/v1/lists and uploaded by an independent researcher into REDCap prior to enrolment.

### 2.6 Surgical technique

Patients underwent standard phacoemulsification cataract surgery with IOL, either partial‐RoF narrow or partial‐RoF extend IOLs. The surgeon received IOL details from a local server file, including patient name, unique Danish Civil Registration Number, and specified IOL type and power. Pupil dilation was achieved using topical phenylephrine 10% and tropicamide 1%. Topical anesthesia with oxybuprocaine 0.4% was administered, followed by ocular surface disinfection with povidone‐iodine. A 2.4 mm clear corneal main incision and a 1.0 mm side‐port incision were created. Intracameral lidocaine 1% was injected, followed by the use of a viscoelastic agent (Healon, Johnson & Johnson, in the right eye; Eyefill C, Bausch & Lomb, in the left eye). Subsequently, capsulorhexis, hydrodissection, phacoemulcification and implantation of the foldable IOL via an injector were performed. At the end of the surgery, intracameral cefuroxime (2.5 mg/mL) was administered for prophylaxis. Postoperatively, patient received anti‐inflammatory treatment with Diclofenac 1 mg/mL administered three times daily for 3 weeks.

Surgeries were performed by experienced surgeons, defined as those who had completed at least 1000 cataract surgeries within the preceding 2 years.

### 2.7 Withdrawal, discontinuation of trial intervention and ancillary and post‐trial care

Patients were withdrawn upon consent withdrawal or significant operative complications. Monovision patients experiencing binocular visual discomfort from anisometropia over 6 months were offered individualized interventions, including photorefractive keratectomy, sulcoflex IOL implantation or a subsidy for spectacles.

### 2.8 Statistical analysis

The power calculation for this pilot trial drew on Catquest questionnaire outcomes (Galvis et al., 2022; Khadka et al., 2016; Lundstrom et al., 2011; Nielsen et al., 2016) from emmetropic patients (partial‐RoF narrow IOLs) and from patients with full‐RoF continuous IOL implantation, as no direct comparison between emmetropic, mini‐monovision, monovision and partial‐RoF extend IOL patients was available in the literature. Postoperative Rasch‐transformed Catquest scores were used for this analysis, which we considered appropriate because pre‐ and postoperative scores show only a weak correlation. Based on these data, detecting a 0.24‐point higher postoperative Rasch‐transformed Catquest score with 80% power and a 5% significance level required 100 patients per group.

All statistical analyses and reportings were performed using SAS Enterprise Guide version 8.4 or higher (SAS Institute Inc., Cary, NC, USA).

Parametric variables were described with mean and standard deviation (SD).

We computed a Rasch‐transformed Catquest‐7SF score for each patient using the IRT procedure. We only transformed scores with two or fewer missing values out of the seven items.

Group comparisons were performed using a linear model (proc glm), with the emmetropia group as the control, as emmetropia is the standard postoperative target at our department. Correlations and associations were assessed using proc. corr, computing Pearson correlation coefficients (Pearson's *r*) and Spearman's correlation coefficients (Spearman's *ρ*), respectively. An alpha value below 0.05 was defined as statistically significant.

## 3 RESULTS

### 3.1 Demographics and baseline characteristics

A total of 191 patients were included, substantially fewer than the target sample size of 400. Recruitment was terminated earlier than planned: 83% of eligible patients declined participation, patient volume was significantly reduced during the COVID‐19 pandemic and a national nurses' strike further limited screening opportunities.

Of the 191 patients included, three patients were not randomized: two declined to participate, and one died prior to randomization. Thus, 188 patients were randomized at baseline in the four groups: emmetropia (*N*: 49), mini‐monovision (*N*: 46), monovision (*N*: 47) and partial‐RoF extend (*N*: 46). The mean age was 74.4, 74.9, 74.6, and 75.1 years, respectively. The gender distribution was consistent across groups, with approximately 37% male and 63% female patients. Eight patients (4.26%) were excluded or withdrew after randomization: four due to surgical complications, two died, one was unable to participate in the final follow‐up due to sequelae from an apoplexy and one declined further participation. This left 180 patients for the primary analysis: emmetropia (*N*: 49), mini‐monovision (*N*: 43), monovision (*N*: 45) and partial‐RoF extend (*N*: 43) (see Figure 1 for a CONSORT flow diagram).

**FIGURE 1:**
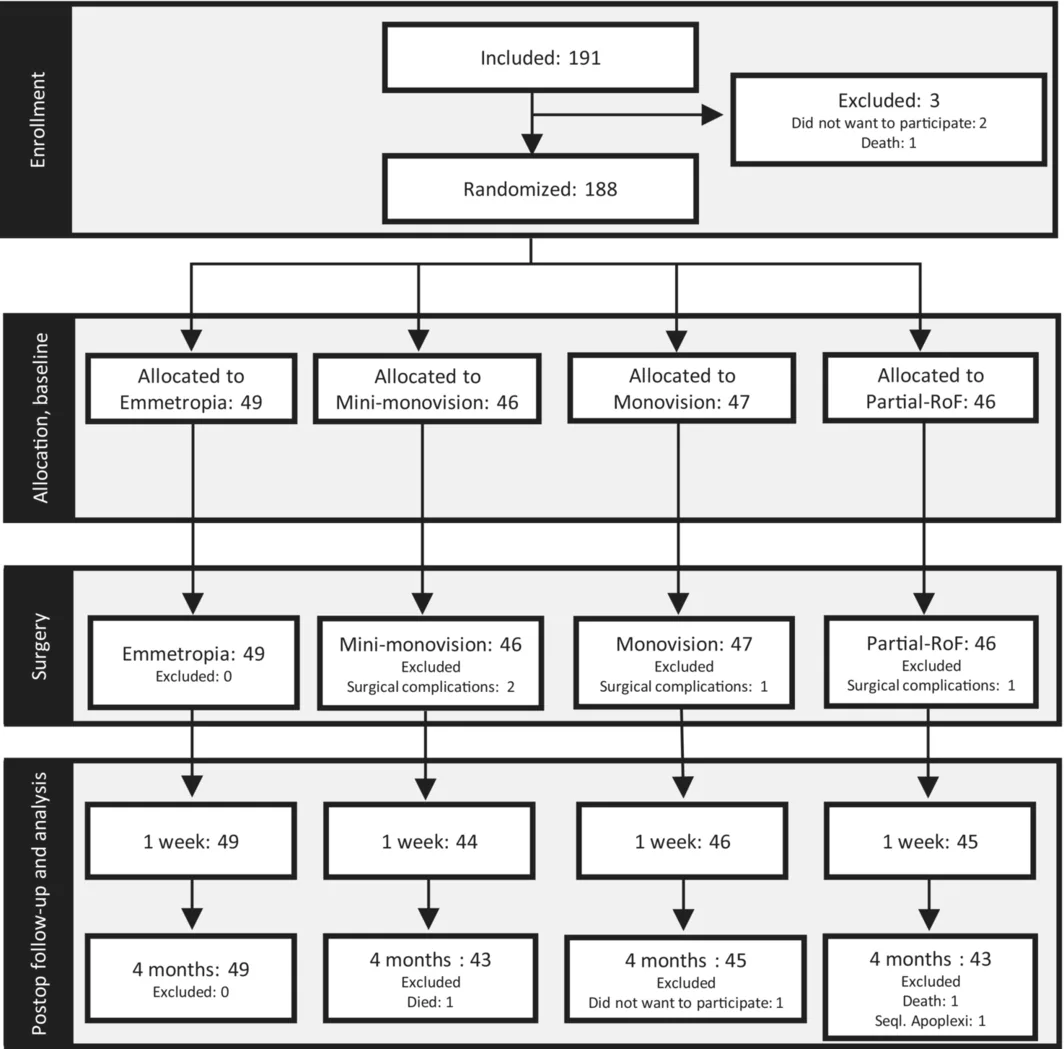
CONSORT flow diagram. Patients underwent same‐day bilateral phacoemulsification and were randomized (1:1:1:1) to: (1) emmetropia (emmetropia in both eyes; partial‐range of field (RoF) narrow intraocular lenses (IOLs)), (2) mini‐monovision (emmetropia in the dominant eye, −1.25 diopters (D) in the non‐dominant; partial‐RoF narrow IOLs), (3) monovision (emmetropia in the dominant eye, −2.50 D in the non‐dominant; partial‐RoF narrow IOLs) or (4) partial‐RoF extend (partial‐RoF) (target minimal hypermetropia (+0.01 to +0.25) in the dominant eye and minimal myopia (−0.01 to −0.25) in the non‐dominant eye; partial‐RoF extend IOLs). Excluded = participants who were randomized but met one or more exclusion criteria; 1 week = 1 week postoperative; 4 months = 4 months postoperative; seql. = sequela.

Table 1 shows the patients' baseline characteristics. Mean aniseikonia was 1.61% ± 2.66%, 1.35% ± 1.96%, 1.57% ± 2.51% and 1.33% ± 2.32% for the emmetropia, mini‐monovision, monovision and partial‐RoF extend groups, respectively. VA was impaired at all distances. Mean binocular UCDVA was logMAR 0.48 ± 0.22, 0.43 ± 0.24, 0.47 ± 0.23 and 0.43 ± 0.25 in the respective groups. No statistically significant differences were found among the four groups for any of the evaluated parameters.

**TABLE 1:** Baseline characteristics.

Parameter	Emmetropia (*N*: 49)	Mini‐monovision (*N*: 46)	Monovision (*N*: 47)	Partial‐RoF (*N*: 46)
Age (year), mean (SD)	74.4 (4.72)	74.9 (4.87)	74.6 (4.63)	75.1 (5.48)
Male/female (%)	38.8/61.2	37.0/63.0	36.2/63.8	37.0/63.0
SE refractive error (D), mean (SD)				
Dominant eye	0.45 (1.97)	0.83 (1.86)	0.52 (2.01)	0.45 (1.78)
Nondominant eye	0.42 (1.98)	0.48 (2.15)	0.56 (1.91)	0.59 (1.99)
Anisometropia (D), mean (SD)	0.69 (0.59)	0.74 (0.62)	0.66 (0.69)	0.57 (0.55)
Aniseikonia (%), mean (SD)	1.61 (2.66)	1.35 (1.96)	1.57 (2.51)	1.33 (2.32)
Stereoacuity (arcsecond), mean (SD)	242 (151)	230 (146)	190 (148)	222 (143)
Distance UCVA (LogMAR), mean (SD)				
Binocular	0.48 (0.22)	0.43 (0.24)	0.47 (0.23)	0.43 (0.25)
Dominant eye	0.59 (0.24)	0.57 (0.27)	0.59 (0.25)	0.52 (0.27)
Nondominant eye	0.60 (0.25)	0.61 (0.27)	0.56 (0.25)	0.56 (0.24)
Intermediate UCVA (LogMAR), mean (SD)				
Binocular	0.47 (0.25)	0.47 (0.23)	0.47 (0.24)	0.44 (0.25)
Dominant eye	0.56 (0.27)	0.60 (0.27)	0.58 (0.27)	0.51 (0.27)
Nondominant eye	0.59 (0.27)	0.59 (0.30)	0.62 (0.25)	0.55 (0.25)
Near UCVA (LogMAR), mean (SD)				
Binocular	0.49 (0.29)	0.46 (0.28)	0.54 (0.25)	0.50 (0.27)
Dominant eye	0.62 (0.31)	0.64 (0.33)	0.67 (0.28)	0.62 (0.27)
Nondominant eye	0.63 (0.33)	0.60 (0.31)	0.68 (0.29)	0.63 (0.29)
Catquest‐7SF (Rasch score), mean (SD)	−0.86 (0.58)	−0.88 (0.50)	−0.78 (0.62)	−0.80 (0.54)
Quality of Vision, frequency, mean (SD)	0.90 (0.50)	0.87 (0.56)	1.00 (0.54)	0.94 (0.47)
Quality of Vision, severity, mean (SD)	0.91 (0.50)	0.92 (0.57)	1.06 (0.56)	0.99 (0.52)
Quality of Vision, bothersome, mean (SD)	0.96 (0.56)	0.91 (0.61)	1.07 (0.63)	0.95 (0.59)
Binocular Visual Function, mean (SD)	1.26 (0.58)	1.24 (0.67)	1.26 (0.68)	1.38 (0.73)

*Note*: Baseline characteristics of patients included in the trial.

Abbreviations: *N*, number of patients; Partial‐RoF, partial‐range of field (RoF) extend lens; SD, standard deviation; SE, spherical equivalent; UCVA, uncorrected visual acuity.

### 3.2 Visual function and spectacle use

Table 2 shows the postoperative outcomes. Postoperative mean anisometropia was 0.25 ± 0.27 D, 1.08 ± 0.44 D, 2.26 ± 0.52 D and 0.51 ± 0.41 D for emmetropia, mini‐monovision, monovision and partial‐RoF extend, respectively. Corresponding mean total aniseikonia NAT was 0.65% ± 1.37%, 1.16% ± 1.76%, 1.46% ± 2.05% and 1.06% ± 1.98% and stereoacuity was 97 ± 82 arcsecond (″), 88 ± 82″, 142 ± 127″ and 126 ± 127″. Statistical analysis showed a significant difference in anisometropia among groups, with the monovision group demonstrating significantly higher aniseikonia and worse stereoacuity compared to the emmetropia group (*p* = 0.03; *p* = 0.04). Mean postoperative contrast sensitivity ranged from 1.64 to 1.68 log contrast sensitivity, with no significant differences between groups (*p* > 0.1) (Table 2).

**TABLE 2:** Postoperative results.

Parameter/group	Mean (SD)	*p* value
SE refractive error (D)		
Dominant eye		
Emmetropia	−0.05 (0.41)	ref.
Mini‐monovision	−0.03 (0.46)	0.87
Monovision	−0.05 (0.31)	0.10
Partial‐RoF	−0.03 (0.42)	0.80
Nondominant eye		
Emmetropia	−0.06 (0.33)	ref.
Mini‐monovision	−1.10 (0.47)	<0.0001
Monovision	−2.32 (0.51)	<0.0001
Partial‐RoF	−0.40 (0.57)	0.0009
Anisometropia (D)		
Emmetropia	0.25 (0.27)	ref.
Mini‐monovision	1.08 (0.44)	<0.0001
Monovision	2.26 (0.52)	<0.0001
Partial‐RoF	0.51 (0.41)	0.004
Aniseikonia (%)		
Emmetropia	0.65 (1.37)	ref.
Mini‐monovision	1.16 (1.76)	0.18
Monovision	1.46 (2.05)	0.03
Partial‐RoF	1.06 (1.98)	0.29
Stereoacuity (arcsecond)		
Emmetropia	97 (82)	ref.
Mini‐monovision	88 (82)	0.69
Monovision	142 (127)	0.04
Partial‐RoF	126 (127)	0.19
Contrast sensitivity (Log CS)		
Emmetropia	1.67 (0.14)	ref.
Mini‐monovision	1.68 (0.13)	0.82
Monovision	1.64 (0.12)	0.24
Partial‐RoF	1.66 (0.12)	0.69

*Note*: Postoperative outcomes in the included patients.

Abbreviations: CS, contrast sensitivity; Partial‐RoF, partial‐range of field (RoF) extend lens; ref., reference group; SD, standard deviation; SE, spherical equivalent.

All patients achieved binocular best corrected distance VA (BCDVA) of logMAR 0.12 or better (range 0.12 to −0.26). Binocular postoperative uncorrected distance VA (UCDVA) for emmetropia, mini‐monovision, monovision and partial‐RoF extend was logMAR −0.01 ± 0.09, 0.05 ± 0.10, 0.08 ± 0.12, and 0.05 ± 0.10, respectively, with the emmetropia group performing significantly better compared to the other three groups (*p* = 0.01; *p* < 0.0001; *p* = 0.02) (Figure 2). This corresponds to the finding of statistically significant lower spectacle use for driving in the emmetropia group compared to mini‐monovision and partial‐RoF extend groups (*p* = 0.01; *p* = 0.03) (Table 3).

**FIGURE 2:**
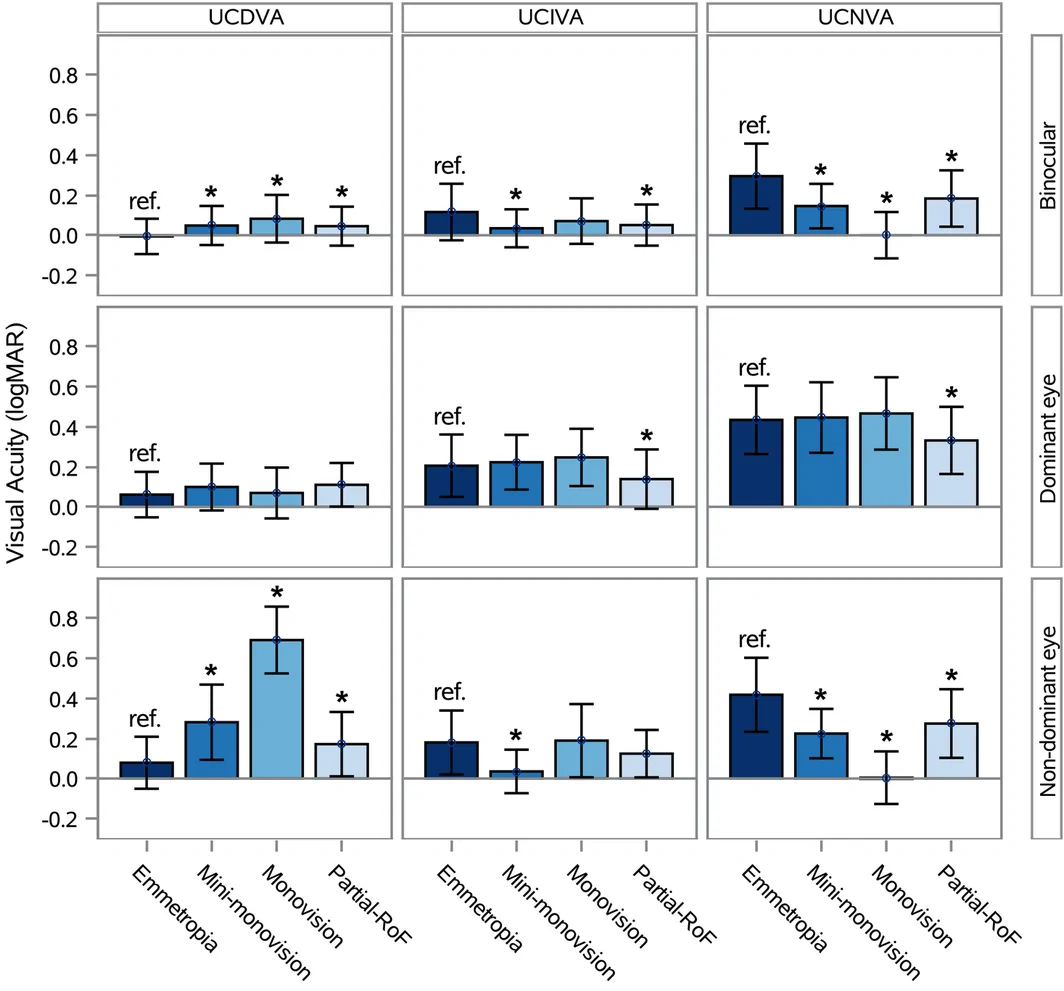
Uncorrected visual acuity at distance, intermediate and near, measured binocularly and in the dominant and non‐dominant eyes. The mean visual acuities are represented by dots, and the vertical error bars represent the standard deviations; ref. = reference group; * = significantly different from the reference group; partial‐RoF = partial‐range of field (RoF) extend lens; UCDVA = uncorrected distance visual acuity; UCIVA = uncorrected intermediate visual acuity; UCNVA = uncorrected near visual acuity.

**TABLE 3:** Postoperative spectacle use (via PRO).

Parameter/group	*N*	Never, %	Occasionally, %	Frequently, %	All the time, %	*p* value
Driving during day						
Emmetropia	34	94.2	2.9	2.9	0.0	ref.
Mini‐monovision	38	84.2	2.6	5.3	7.9	0.01
Monovision	37	83.8	8.1	5.4	2.7	0.11
Partial‐RoF	34	79.4	3.0	8.8	8.8	0.03
Seeing the computer monitor						
Emmetropia	44	20.4	29.6	11.4	38.6	ref.
Mini‐monovision	39	53.9	12.8	20.5	12.8	0.01
Monovision	42	66.7	14.3	9.5	9.5	0.0005
Partial‐RoF	41	48.8	19.5	14.6	17.1	0.05
Watching TV						
Emmetropia	47	70.2	12.8	6.4	10.6	ref.
Mini‐monovision	41	78.0	12.2	4.9	4.9	0.32
Monovision	44	70.4	11.4	9.1	9.1	0.91
Partial‐RoF	43	72.1	4.7	11.6	11.6	0.57
Reading newspaper size print						
Emmetropia	49	16.3	22.5	10.2	51.0	ref.
Mini‐monovision	42	35.7	11.9	14.3	38.1	0.06
Monovision	43	67.4	16.3	4.7	11.6	<0.0001
Partial‐RoF	43	37.2	18.6	23.3	20.9	0.01
Using your mobile phone						
Emmetropia	49	10.2	24.5	14.3	51.0	ref.
Mini‐monovision	43	46.5	16.3	13.9	23.3	0.0002
Monovision	44	72.7	11.4	2.3	13.6	<0.0001
Partial‐RoF	43	44.2	18.6	20.9	16.3	<0.0001

*Note*: Postoperative spectacle use in the included patients.

Abbreviations: *N*, number of patients; Partial‐RoF, partial‐range of field (RoF) extend lens; ref., reference group.

Binocular postoperative uncorrected intermediate VA (UCIVA) was logMAR 0.12 ± 0.14, 0.03 ± 0.09, 0.07 ± 0.11, 0.05 ± 0.10 for emmetropia, mini‐monovision, monovision and partial‐RoF extend, respectively. Mini‐monovision and partial‐RoF extend demonstrated significantly better UCIVA compared to emmetropia, with monovision showing borderline better UCIVA compared to emmetropia (Figure 2). Correspondingly, spectacle use for computer viewing was significantly lower in mini‐monovision, monovision and partial‐RoF extend groups compared to emmetropia, with patients in the monovision group demonstrating the lowest usage unequivocally (*p* = 0.0005) between the groups (Table 3).

Binocular postoperative uncorrected near VA (UCNVA) for emmetropia, mini‐monovision, monovision and partial‐RoF extend was logMAR 0.29 ± 0.16, 0.15 ± 0.11, 0.00 ± 0.12, and 0.18 ± 0.14, with emmetropia performing significantly worse compared to the other groups (Figure 2). Correspondingly, spectacle use for reading was significantly higher in the emmetropia group than in the monovision (*p* < 0.0001) and partial‐RoF extend (*p* = 0.01) groups. Spectacle use for mobile phone tasks was also significantly higher in the emmetropia group compared with mini‐monovision (*p* = 0.0002), monovision (*p* < 0.0001) and partial‐RoF extend (*p* < 0.0001) (Table 3).

Complete spectacle independence was not achieved across any entire group; 38 patients were completely spectacle independent at distance, intermediate and near: two in the emmetropia group, nine in both the mini‐monovision and partial‐RoF extend groups and 18 in the monovision group (see Figure 3 for the percentage of patients in each group who were spectacle independent for different activities).

**FIGURE 3:**
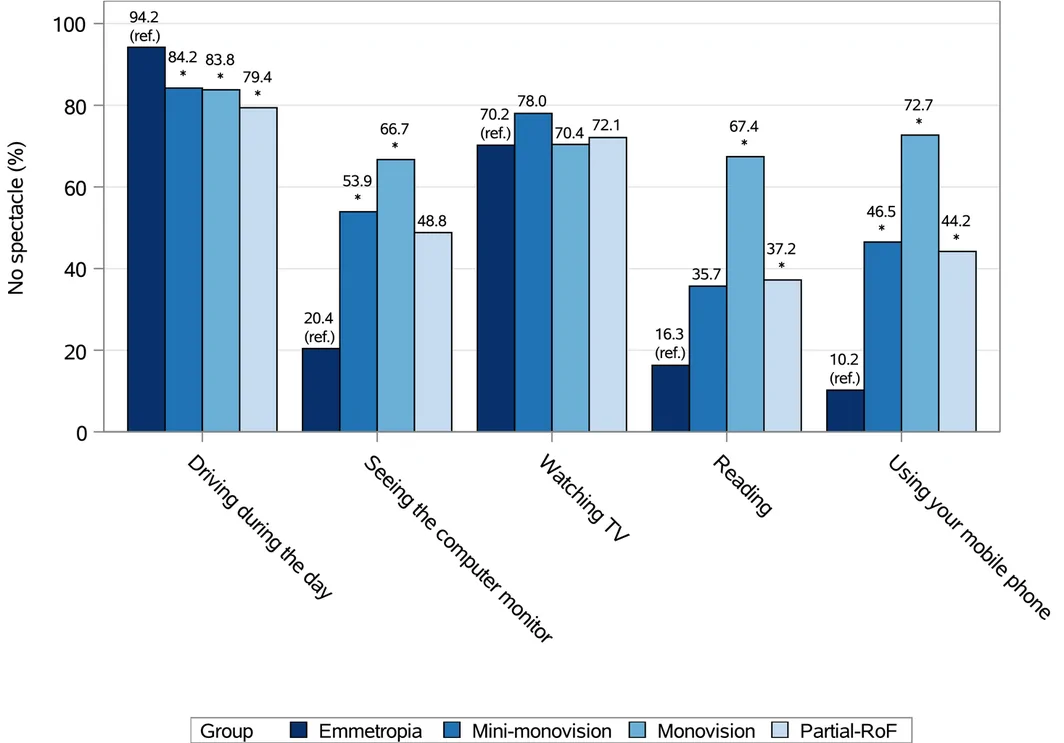
Percentage of patients not using spectacles across different activities; ref. = reference group; * = significantly different from the reference group; partial‐RoF = partial‐range of field (RoF) extend lens.

### 3.3 Patient‐reported outcomes

All four groups reported improvement in PROM scores postoperatively compared to preoperatively, with no statistically significant differences in ∆‐scores between groups. Patients in the monovision group demonstrated a non‐significant trend to higher ∆‐scores in the frequency and bothersome subscales of QoV, consistent with their significantly higher halo frequency compared to patients in the emmetropia group (Tables 4 and 5). Table 4 shows the ∆‐scores of Catquest‐7SF, QoV and BVF.

**TABLE 4:** Delta questionnaire scores.

Parameter/group	Mean ∆ (SD)	*p* value
Catquest‐7SF (Rasch score)		
Emmetropia	1.33 (0.67)	ref.
Mini‐monovision	1.47 (0.59)	0.35
Monovision	1.17 (0.85)	0.30
Partial‐RoF	1.42 (0.73)	0.55
Quality of vision, frequency		
Emmetropia	−0.60 (0.49)	ref.
Mini‐monovision	−0.61 (0.56)	0.94
Monovision	−0.47 (0.68)	0.25
Partial‐RoF	−0.60 (0.48)	0.98
Quality of vision, severity		
Emmetropia	−0.59 (0.50)	ref.
Mini‐monovision	−0.65 (0.57)	0.61
Monovision	−0.52 (0.69)	0.56
Partial‐RoF	−0.63 (0.55)	0.69
Quality of vision, bothersome		
Emmetropia	−0.68 (0.56)	ref.
Mini‐monovision	−0.69 (0.60)	0.97
Monovision	−0.57 (0.75)	0.39
Partial‐RoF	−0.67 (0.60)	0.90
Binocular visual function		
Emmetropia	−0.68 (0.57)	ref.
Mini‐monovision	−0.68 (0.47)	0.97
Monovision	−0.51 (0.70)	0.17
Partial‐RoF	−0.84 (0.64)	0.19

*Note*: Delta scores of the three questionnaires used in the trial among the included patients.

Abbreviations: ∆, delta = postoperative value − preoperative value; Partial‐RoF, partial‐range of field (RoF) extend lens; ref., reference group; SD, standard deviation.

**TABLE 5:** Postoperative glare, halos and starbursts symptoms (via QoV).

Parameter/group	*N*	Never, %	Occasionally, %	Quite often, %	Vere often, %	*p* value
Glare						
Emmetropia	49	38.8	55.1	4.1	2.0	ref.
Mini‐monovision	43	44.2	48.8	0.0	7.0	0.98
Monovision	45	35.6	44.4	11.1	8.9	0.12
Partial‐RoF	43	34.9	62.8	2.3	0.0	0.90
Halos						
Emmetropia	49	67.4	30.6	2.0	0.0	ref.
Mini‐monovision	43	69.8	18.6	9.3	2.3	0.52
Monovision	45	48.9	40.0	4.4	6.7	0.02
Partial‐RoF	43	62.8	32.6	2.3	2.3	0.52
Starbursts						
Emmetropia	49	59.2	36.7	4.1	0.0	ref.
Mini‐monovision	43	69.8	18.6	11.6	0.0	0.83
Monovision	45	51.1	33.3	11.1	4.5	0.09
Partial‐RoF	43	48.8	46.5	4.7	0.0	0.45

*Note*: Postoperative glare, halos and starbursts in included patients.

Abbreviations: *N* = number of patients; Partial‐RoF, partial‐range of field (RoF) extend lens; ref. = reference group.

Binocular visual complaints, assessed via BVF, were generally minimal and mild, with no statistically significant difference among the groups. However, a non‐significant trend was observed with patients in the monovision group reporting the highest ∆BVF score (−0.51 ± 0.70) and patients in the partial‐RoF extend group reporting the lowest (−0.84 ± 0.64).

Of the patients using spectacles, 76% reported being ‘not bothered at all’ or ‘very little bothered’ by needing to wear spectacles for some activities, as assessed using the PRO questionnaire.

### 3.4 Satisfaction

Single items on different aspects of patient satisfaction were assessed using the PRO questionnaire. No statistically significant difference between groups was found for the item regarding overall patient satisfaction with vision after surgery, although the monovision group was borderline significantly less satisfied than the emmetropia group (*p* = 0.05). Being ‘very satisfied’ was reported in 75.1% of the emmetropia group, 76.7% in the mini‐monovision group, 62.2% in the monovision group and 67.4% in the partial‐RoF extend group. Regarding the item, whether vision compared to the expected ‘much better than expected’ was reported in 38.8% of emmetropia, 58.1% of mini‐monovision, 28.9% of monovision and 51.2% of the partial‐RoF extend group. Monovision was statistically significantly higher compared to emmetropia (*p* = 0.01).

The likelihood of patients referring a friend or family member for the same surgery with the identical implant was also evaluated using the PRO questionnaire. High referral likelihood was observed in the emmetropia (73.5%), mini‐monovision (72.1%) and partial‐RoF extend (76.7%) groups, but lower in the monovision group (55.6%). The difference between the emmetropia and monovision was statistically significant (*p* = 0.03).

The likelihood of patients referring a friend or family member for the same surgery was significantly correlated with the delta of Catquest‐7SF, QoV and BVF (Pearson's *r*: 0.26–0.37; *p* ≤ 0.0004).

### 3.5 PROM correlations

Improvement in PROM scores postoperatively measured by ∆Catquest‐7SF, ∆QoV frequency and ∆BVF significantly correlated with better postoperatively non‐dominant eye UCDVA (Pearson's *r*: 0.15–0.18; *p*‐value range: 0.02–0.04). Additionally, ∆Catquest‐7SF was associated with preoperative stereoacuity (Spearman's *ρ* = 0.24; *p* = 0.002) (for all groups). Spearman's *ρ* = 0.35; *p* = 0.0008 (for the mini‐monovision and monovision groups).

No additional correlations were identified between ∆PROMs and VA, spectacle use, anisometropia, aniseikonia or contrast sensitivity.

### 3.6 Safety

Adverse events were assessed at each visit, and medical records were reviewed for hospitalizations related to falls, fractures, dizziness or traffic accidents that might be related to visual function. Perioperative complications occurred in three patients: iris prolapse (mini‐monovision), zonular weakness with vitreous prolapse (monovision) and aqueous misdirection (partial‐RoF extend). One postoperative retinal detachment occurred (mini‐monovision). All four were withdrawn. Three monovision patients (6.7%) required reoperation due to binocular visual complaints (all three underwent reoperation with Sulcoflex implantation after the final follow‐up, following an adaptation period of at least 6 months). No significant differences were observed in hospitalizations related to falls, fractures, dizziness or traffic accidents.

## 4 DISCUSSION

This randomized controlled trial evaluated PROMs and visual function following cataract surgery using one of four refractive strategies: emmetropia, mini‐monovision, monovision with partial‐RoF narrow IOLs and partial‐RoF extend IOLs.

All patients achieved a binocular best‐corrected distance VA of logMAR 0.12 or better (range 0.12 to −0.26), which is reassuring when selecting a refractive strategy. The overall VA outcomes were consistent with previous reports, with the emmetropic group achieving the best binocular UCDVA, the mini‐monovision and partial‐RoF extend groups performing best in binocular UCIVA and the monovision group performing best in binocular UCNVA (Bala et al., 2022; Goldberg, 2003; Hogarty et al., 2018; Hovanesian et al., 2022; Rodov et al., 2019; van Amelsfort et al., 2022). Consistent with earlier findings, spectacle independence for distance was highest in the emmetropic group, while independence from reading glasses was greatest in the monovision group (Goldberg, 2001, 2003; Greenbaum, 2002; Rodov et al., 2019). However, a higher‐than‐expected rate of spectacle use was observed across all distances, particularly for driving, where patients in the mini‐monovision and partial‐RoF extend groups had the highest numerical spectacle use. This may reflect residual myopia in the non‐dominant eye in these two groups. The monovision group demonstrated the highest rate of spectacle independence, with 40% of patients being spectacle independent.

Postoperative aniseikonia was significantly higher and stereoacuity poorer in the monovision group compared with the emmetropic group, consistent with the rule of thumb (1 diopter of spectacle‐corrected anisometropia results in 1% of aniseikonia) and previous studies (Berens & Bannon, 1963; Campos & Enoch, 1980; Katsumi et al., 1986; Ogle, 1950). Despite this, postoperative binocular visual symptoms and overall PROM scores were similar across groups, although the monovision group showed a non‐significant trend toward greater symptom frequency.

Subjective QoV, assessed using the QoV questionnaire, demonstrated that halos occurred significantly more frequently in the monovision group than in the other three groups. This finding aligns with previous reports that glare, halos and starburst symptoms are generally infrequent and mild with the AcrySof IQ Vivity IOL, whereas halos are more common among monovision patients (Bala et al., 2022; Evans, 2007; Hovanesian et al., 2022; Run Johannsdottir & Stelmach, 2001; van Amelsfort et al., 2022).

All PROMs (QoV, Catquest and BVF) showed significant improvements from baseline across groups, consistent with previous studies (Britton et al., 2022; Hovanesian et al., 2022; Krarup et al., 2020; Rodov et al., 2019). Despite clear variations in spectacle independence, stereoacuity and corrected and uncorrected VA across the four refractive strategies, no statistically significant intergroup differences were detected in delta PROM scores. Several factors likely contributed to the lack of statistically significant between‐group differences. It is possible that the substantial vision improvement inherent to cataract surgery itself, regardless of refractive strategy, is the primary driver of patient satisfaction, effectively equalizing outcomes across groups and rendering the additional benefit of spectacle reduction of secondary importance.

This pronounced vision gain may have caused score clustering at the upper end of the PROM scales, limiting the ability to detect subtle but clinically relevant differences between groups. This ceiling effect was likely further compounded by instrument limitations, as many PROM items were generic in nature despite the use of two Rasch‐validated questionnaires specifically designed for cataract and refractive populations. Response bias, whereby patients reported satisfaction with any degree of postoperative improvement irrespective of the specific refractive outcome, may have further diminished the variability required for group discrimination.

Consistent with this interpretation, spectacle use did not correlate with delta PROM scores, and 76% of spectacle‐wearing patients reported no or minimal inconvenience associated with wearing spectacles for certain activities. This likely reflects the characteristics of the study population: public hospital cataract patients referred primarily for functional visual rehabilitation rather than spectacle independence. Although patients were informed about the potential for spectacle reduction, they were not asked to prioritize spectacle independence, and they remained masked to their allocated strategy until after PROM completion. Greater between‐group differences might be expected in a population actively seeking spectacle independence.

The monovision group achieved good uncorrected binocular vision at near and intermediate distances, while uncorrected distance vision was lower than in the other groups. BVF scores did not worsen significantly, and spectacle independence rates were high across all distances, with 40% completely spectacle independent.

Despite these outcomes, only 62.2% reported being ‘very satisfied’ and 55.6% were ‘very likely’ to recommend the procedure—lower than in the other groups. This may suggest that this specific questionnaire item is more sensitive to aspects of visual discomfort or binocular imbalance associated with monovision, potentially identifying intolerance that is not fully captured by the overall PROM score. Notably, this item correlated with all three delta PROM scores.

Three patients (6.7%) required reoperation due to binocular visual complaints, whereas none did in the other groups. The acceptability of this rate is subjective and highlights the importance of careful patient selection, ideally including preoperative tolerance testing (e.g., contact lens simulation), as well as thorough counselling regarding the trade‐offs between spectacle independence and the potential for binocular visual discomfort.

Correlation analysis identified two predictors of higher postoperative improvement in PROMs: poorer preoperative stereoacuity and better uncorrected distance VA in the non‐dominant eye. It seems reasonable that a poorer stereoacuity preoperatively might result in a more pronounced amelioration, which can be reflected in the PROMs. To our knowledge, the relationship between non‐dominant eye VA and delta PROMs has not been previously described. This finding seems plausible, as poor tolerance to blur in the non‐dominant eye and insufficient blur suppression may contribute to dissatisfaction, consistent with previous studies (Collins & Goode, 1994; Evans, 2007). Collectively, these results suggest that individuals with strong binocular vision may depend more on binocular fusion, making them less tolerant of anisometropia and more prone to visual discomfort.

The strengths of this randomized trial include stratified randomization, assessment of several Rasch‐validated PROMs, binocular function assessment, assessor consistency, and long‐term follow‐up. Limitations include the pilot sample size (45 versus the planned 100), which may leave the study underpowered to exclude small inter‐treatment group differences in PROMs. The absence of statistically significant differences across all four PROMS and refractive strategies, therefore, cannot definitely rule out smaller clinically relevant effects. However, looking at all four PROMs and four groups, none of the comparisons showed meaningful trends, which support the interpretation that either patients experienced genuinely comparable outcomes, or the vision gain from the cataract surgery outshone refractive differences, rendering standard cataract PROMs insensitive to compare refractive results in routine cataract patients. Another limitation is the change in the partial‐RoF narrow IOL model during the study period. However, the differences between the two IOLs are minor and unlikely to have influenced visual outcomes meaningfully. Also, Alcon AcrySof IQ Vivity IOL incorporates ultraviolet and blue‐light filtering, unlike the other IOLs. Additional limitations include the use of unvalidated questionnaires and the use of patients' own spectacles in contrast sensitivity measurements.

In conclusion, all four refractive strategies resulted in significant improvements in patient‐reported outcomes and visual function, with no statistically significant differences between groups. The three strategies aimed at reducing spectacle dependence did not confer additional PROM benefits beyond the visual improvement achieved through cataract surgery itself. Mini‐monovision and partial‐RoF extend yielded comparable outcomes across visual performance, spectacle independence and safety. Monovision offered the greatest spectacle independence but at the cost of reduced binocular visual quality, lower patient endorsement and a 6.7% reoperation rate due to binocular visual complaints.

## Supporting information


Data S1.
