# Supplementary material for: Patient‐reported outcomes and visual function after four refractive strategies in cataract surgery: A randomized controlled trial
Source: Acta Ophthalmol. 2026 Apr 13;104(8):855–67. doi: 10.1111/aos.70145 (PMC13751849; doi:10.1111/aos.70145)
Supplement: Supplementary file 1 [file AOS-104-855-s001.docx]

## Supporting Information 1. Binocular Visual Function

The following questions address any visual discomfort you may experience. Please respond based on how you see when wearing your glasses (if you use glasses).

|  |  | Never | Occasionally | Sometimes | Quite often | Always |
| --- | --- | --- | --- | --- | --- | --- |
| **1** | Do you experience headaches? |  |  |  |  |  |
| **2** | Do your eyes feel strained, with sensations such as fatigue, soreness, burning, pain, or pulling? |  |  |  |  |  |
| **3** | Are you sensitive to light? |  |  |  |  |  |
| **4** | Do you experience reading difficulties? |  |  |  |  |  |
| **5** | Do you close one eye when you read? |  |  |  |  |  |
| **6** | Do you feel anxious because of your vision? |  |  |  |  |  |
| **7** | Do you experience double vision? |  |  |  |  |  |
| **8** | Do you experience nausea? |  |  |  |  |  |
| **9** | Do you feel that you read slowly? |  |  |  |  |  |
| **10** | Do you feel dizzy? |  |  |  |  |  |
| **11** | Do you feel tired? |  |  |  |  |  |
| **12** | Do you feel that your perception of objects and space around you is distorted (altered in shape)? |  |  |  |  |  |
|  | *Completed by the ophthalmology department* | ____x0 | ____x1 | ____x2 | ____x3 | ____x4 |

Please answer the following questions regarding how your eyes feel when you read or look at distant objects.
